# General Regression and Representation Model for Classification

**DOI:** 10.1371/journal.pone.0115214

**Published:** 2014-12-22

**Authors:** Jianjun Qian, Jian Yang, Yong Xu

**Affiliations:** 1 School of Computer Science and Engineering, Nanjing University of Science and Technology, Nanjing, 210094, China; 2 Bio-Computing Research Center, Shenzhen Graduate School, Harbin Institute of Technology, Shenzhen, 518055, China; Shanghai Jiaotong University, China

## Abstract

Recently, the regularized coding-based classification methods (e.g. SRC and CRC) show a great potential for pattern classification. However, most existing coding methods assume that the representation residuals are uncorrelated. In real-world applications, this assumption does not hold. In this paper, we take account of the correlations of the representation residuals and develop a general regression and representation model (GRR) for classification. GRR not only has advantages of CRC, but also takes full use of the prior information (e.g. the correlations between representation residuals and representation coefficients) and the specific information (weight matrix of image pixels) to enhance the classification performance. GRR uses the generalized Tikhonov regularization and K Nearest Neighbors to learn the prior information from the training data. Meanwhile, the specific information is obtained by using an iterative algorithm to update the feature (or image pixel) weights of the test sample. With the proposed model as a platform, we design two classifiers: basic general regression and representation classifier (B-GRR) and robust general regression and representation classifier (R-GRR). The experimental results demonstrate the performance advantages of proposed methods over state-of-the-art algorithms.

## Introduction

As well known, the nearest neighbor classifier (NN) is one of the most popular classifiers due to its simplicity and efficiency. However, NN just uses one training sample to represent test sample. To address this problem, the nearest feature line (NFL) uses two training samples of each class to represent test sample [1]. The nearest feature plane (NFP) applies three samples to represent test sample [2]. Furthermore, some classifiers leverage more training samples for test sample representation, such as the local subspace classifier (LS) [3] and nearest subspace classifier (NS) [4], [27], which represent the test sample via all training samples of each class. Actually, all these methods can be considered as variants of linear regression based methods. To prevent over-fitting, the L_2_-regularizer is generally used in the linear regression model. In the past years, the L_1_-regularizer, which is closely linked to sparse representation, becomes a hot theme in information theory, signal/image processing and related areas. Meanwhile, numerous findings of neuroscience and biology form a physiological base for sparse representation [5]–[7].

Recently, many efforts have been made to apply sparse representation methods to pattern classification tasks, including signal/image classification and face recognition etc. Labusch et al. presented a simple sparse-coding strategy for digit recognition and achieved state-of-the-art results on the MNIST benchmark [8]. Yang et al. addressed the problem of generating a super-resolution (SR) image from a single low-resolution input image via sparse representation [9]. Mairal et al. elaborated a framework for learning multi-scale sparse representations of images with applications to image denoising and inpainting [10]. Yang et al. employed sparse coding instead of vector quantization to capture the significant properties of local image descriptors for image classification [11]. Particularly, Wright et al. introduced a sparse representation based classification (SRC) and successfully applied it to identify human faces with varying illumination changes, occlusion and real disguise [12]. In their method, a test sample image is coded as a sparse linear combination of the training images, and then the classification is achieved by identifying which class yields the least residual. Theodorakopoulos et al. introduced a face recognition method based on sparse representation of facial image patches [37]. Subsequently, Gao et al. proposed Kernel Sparse Representation for image classification and face recognition. KSR actually is the sparse coding technique in a high dimensional feature space via some implicit feature mapping [39]. Yang and Zhang constructed a Gabor occlusion dictionary for SRC to reduce the computation cost by using Gabor feature [13].

Although the newly-emerging SRC shows great potential for pattern classification, it lacks theoretical justification. Yang et al. provided an insight into SRC and analyzed the role of L_1_-optimizer [14]. They think that L_1_-optimizer contains two properties: sparsity and closeness. However, L_0_-optimizer can only achieve the sparsity. Sparsity determines a small number of nonzero representation coefficients and closeness makes the nonzero representation coefficients concentrate on the training samples with the same class label as the given test sample. Wright et al. give an overview of sparse representation for computer vision and pattern recognition [15]. Yang et al. presented a robust regularized coding model to enhance the robustness of face recognition to occlusion, pixel corruption and real disguises [16], [31]. He et al. proposed an effective sparse representation algorithm based on maximum correntropy criterion for robust face recognition [17]. To unify the existing robust sparse regression models: the additive model represented by SRC for error correction and multiplicative model represented by CESR and RSC for error detection, He et al. [38] built a half-quadratic framework by defining different half-quadratic functions. The framework enables to perform both error correction and error detection. Furthermore, He et al. also leverage the half-quadratic framework to address the feature selection and subspace clustering problems [48], [49]. In addition, Zhou et al. incorporated the Markov Random Field model into the sparse representation framework for spatial continuity of the occlusion [40]. Li et al. explored the intrinsic structure of continuous occlusion and proposed the structured sparse error coding (SSEC) model [41]. Ou et al. proposed a novel structured occlusion dictionary learning method for robust face recognition [42]. Apart from these methods, many related tasks have been reported [18]–[20], [32]–[35].

With the widely use of sparse representation for classification, some scholars question the role of sparseness for image classification [21], [22]. Zhang et al. analyzed the working principle of SRC and believed that it is the collaborative representation that improves the image classification accuracy rather than the L_1_-norm sparsity. Consequently, Zhang et al. presented a collaborative representation based classification with regularized least square (CRC) [23]. Compared with SRC, CRC delivers very competitive classification results with little computation time. Subsequently, Yang et al. proposed a relaxed collaborative representation model (RCR) which effectively captures the similarity and distinctiveness of different features for pattern classification [24]. Theodorakopoulos et al. gave a collaborative sparse representation model in dissimilarity space for visual classification tasks [43].

**Figure 1:**
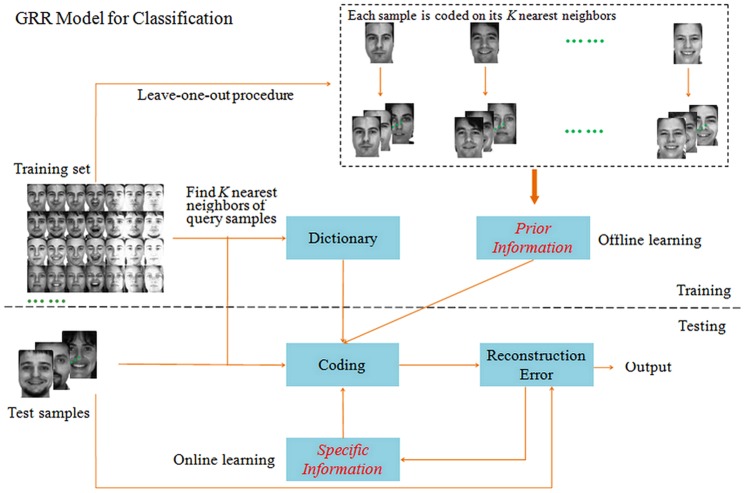
An overview of General Regression and Representation model for classification.

Most of previous works assume that representation residuals are mutually uncorrelated [31]. It's difficult to hold this assumption in real-world applications. Actually, it is common to have data where representation residuals are correlated. Thus, in this paper, we consider to eliminate the correlations between representation residuals and present a novel model named General Regression and Representation (GRR) for pattern classification. GRR mainly aims to take account of the prior information (e.g. the correlations between representation residuals and representation coefficients) and the specific information (weight matrix for each image pixel) so as to enhance the classification performance under different conditions. Specifically, GRR selects one image from training set and finds its K nearest neighbors in rest ones to code the image. In this way, all the training images can be coded on its K nearest neighbors. Subsequently, we calculate the correlations between representation residuals and representation coefficients by virtue of the reconstruction error and representation coefficient of each training image. For each test sample, we apply the iterative algorithm to achieve the weight of each image pixel. The overview of GRR is shown in Fig. 1. Compared with other regression based classification methods, the novelty of the proposed model is threefold:

- First, we take into account the correlations of the representation residuals and develop a general regression and representation model (GRR) for sample coding;
- Second, GRR captures the prior information from the training set via the generalized Tikhonov regularization in conjunction with the K Nearest Neighbor method and leaving-one-out procedure;
- Third, with the GRR model as a platform, we design two classifiers: Basic GRR (B-GRR) and Robust GRR (R-GRR) by combining the prior information and the specific information with different strategies.

To evaluate the proposed model, we finally use four databases which involve different recognition tasks: the CENPARMI dataset for handwritten numerical recognition, the NUST603 dataset for handwritten Chinese character recognition, the AR dataset for face recognition and face occlusion recognition, and the Extended Yale B dataset for face recognition with extreme lighting changes and face recognition with random block occlusion. Experimental results demonstrate the effectiveness of the proposed model.

This paper is the extended version of our conference paper [36]. In this paper, we provide a more in-depth analysis and more extensive experiments on the proposed model.

## Current Methods and Their Problems

### A. Current Methods

Suppose there are *c* known pattern classes. Let 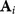 be the matrix formed by the training samples of Class *i*, i.e., 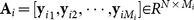, where 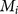 is the number of training samples of Class *i*. Let us define a matrix 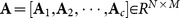, where 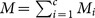. The matrix 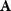 is obviously composed of entire training samples.

#### Sparse Representation based Classification

Given a test sample **y**, we present **y** in a over-complete dictionary whose basis vectors are training samples themselves. i.e., **y** = **Ax**. The sparse solution to **y** = **Ax** can be sought by solving the follow optimization problem:
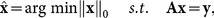
(1)


However, solving the *L*_0_ optimization is NP hard problem. Fortunately, recent researches reveal that *L*_0_ optimization and *L*_1_ optimization are equivalent when the solution is sparse enough. In general, the sparse representation problem can be formulated as: 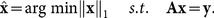
(2)


The problem is equivalent to 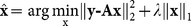.

Then classification rule is:
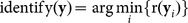
(3)where 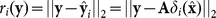, 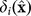 is the nonzero coefficient vector associated with class *i*.

#### Correntropy-based Sparse Representation

Correntropy-based Sparse Representation (CESR) leverages the maximum correntropy criterion to design the classifier for robust face recognition [17]. Similar with SRC, CESR aims to reconstruct a test sample **y** using existing training samples as well as possible. The correntropy-based sparse model is formulated as:
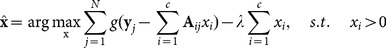
(4)where 
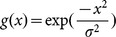
 is a gaussian kernel function. The above nonlinear objective function can be solved by using half-quadratic optimization technique. Then, the test sample is classified to class *i* corresponding to the maximal nonlinear difference between **y** and 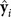, i.e.,
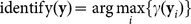
(5)where 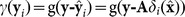 and 
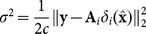
.

#### Robust Sparse Representation

The robust sparse representation problem can be reformulated as the following minimization problem:
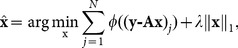
(6)where 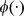 is a robust M-estimator and can be optimized by half-quadratic (HQ) optimization, 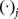 means the *j*-th dimension of input data. In HQ framework, RSR problem can be considered as an iterative regularization problem and applying a number of unconstrained quadratic problems to solve the optimization problem. The classification rule is 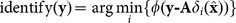
(7)


#### CRC with Regularized Least Square

CRC uses the regularized least square method to represent test sample can lead the similar results to L_1_-norm regularization but with low computation burden. The model is formulated as:
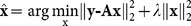
(8)where 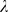 is the regularization parameter. The regularization term can help us to achieve a stable solution. Meanwhile, it also introduces a little sparse constraint to the 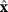, which is much weaker than SRC. The solution of CRC in (8) with regularized least square as follows:
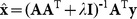
(9)


The classification rule of CRC is similar with SRC. However, 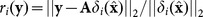. We classify **y** by checking the reconstruction error of each class to yield the classification result.

#### Linear Regression Classification

It's assumed that patterns from the same class lie on a linear subspace. On the basis of this point, LRC represents the test sample image as a linear combination of class-specific training set. There is: 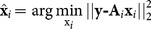
(10)


The solution of 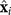 is: 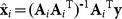
(11)


LRC is made in favor of the class with the minimum distance 
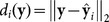
.

### B. Problems

**Figure 2:**
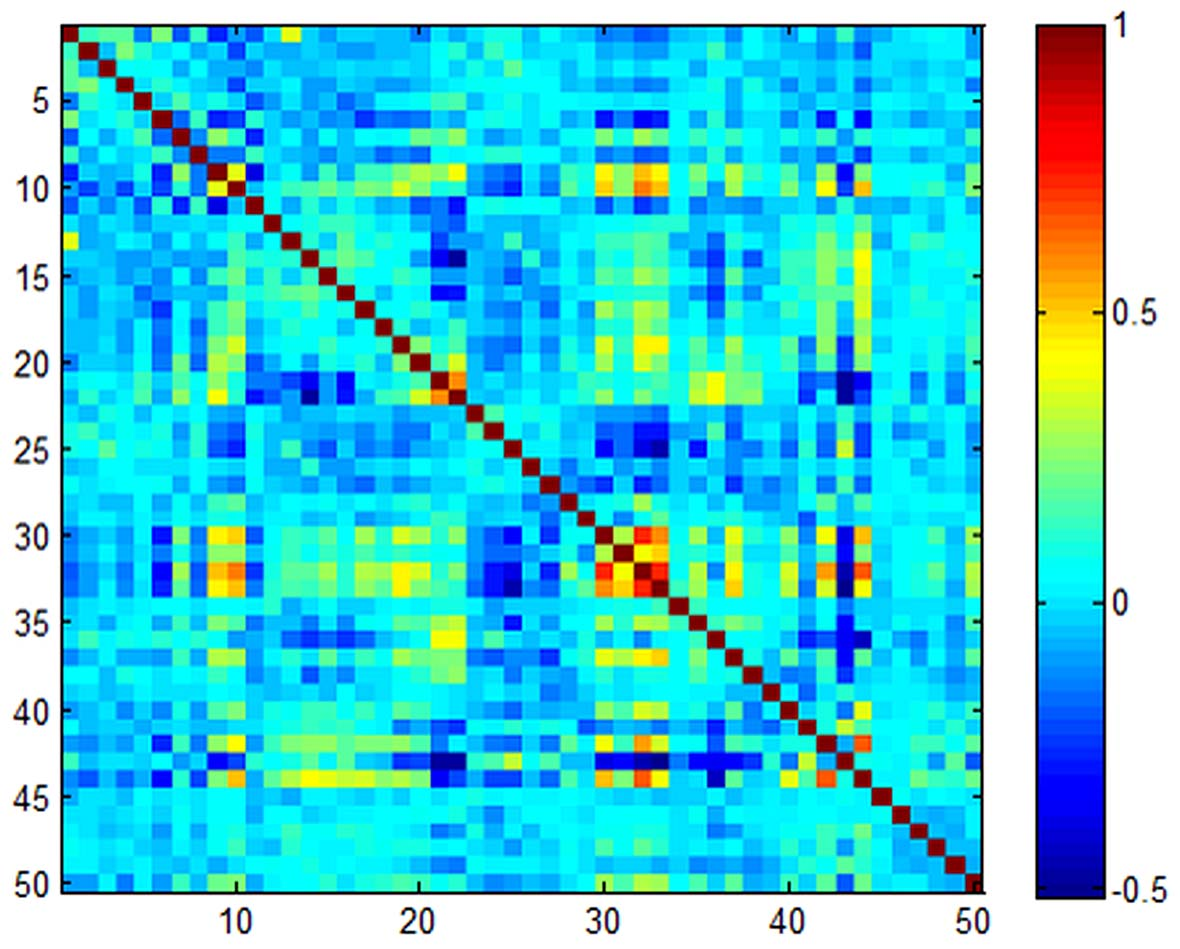
An example shows partial correlation matrix (only select top 50 from 121 ones) of representation residuals.

The most previous works, including RSC, SRC, CRC, CESR et al, assume that the representation residuals are homoskedastic and mutually uncorrelated. In real-world applications, these assumptions do not hold. In particular, when the elements of representation residuals have unequal variances and are correlated, variance of representation residuals is no longer a scalar variance-covariance matrix, and hence there is no guarantee that the least square estimator is the most efficient within the class of linear unbiased estimators [46], [47]. Here, we also give an example to demonstrate this view. Fig. 2 shows the example, where 200 samples of each class are selected from the CENPARMI dataset, each sample is coded on its top 200 neighbors from the rest samples. The correlation matrix map of representation residuals is shown in Fig. 2, from which we can see that these representation residuals are actually correlated.

## General Regression and Representation Model for Classification (GRR)

This section mainly introduces two classifiers: Basic GRR and Robust GRR, which designed with the GRR model as a platform. Basic GRR is built to address the correlation problem of representation residuals in other regression models. Robust GRR is an extended version of the basic GRR model, which provides a mechanism to deal with noises in test samples.

### A. Basic GRR

Let **A** be the matrix formed by the K nearest neighbors of the test sample from training set, M be the number of training samples, and **y** be the test sample. Our model is 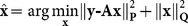
(12)where **P** is the matrix that is introduced to eliminate the correlations between representation residuals (or called reconstruction errors), and **Q** is used to refine the regularization term.

We call above model as the basic general regression and representation (B-GRR). Actually, this model can be reformulated as follows: 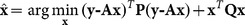
(13)


If **P** and **Q** are known, from the generalized Tikhonov regularization [30], [44], we know there is a close-form solution: 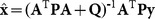
(14)


However, **P** and **Q** are unknown beforehand. We here employ a generative method to estimate these two correlation matrices **P** and **Q** in the training stage. Basically, we assume the representation residual 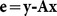 and representation coefficient vector **x** satisfy multivariate normal distributions. Then, **P** can be estimated by using the inverse covariance matrix of **e** [30], [44]. To explain why the matrix **P** can be estimated in this way, we first let **R** be a non-stochastic transformed matrix and ignore the regularization term for the moment. Eq. (11) can be reformulated as 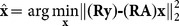
(15)where **Ry** denotes the transformed dependent variable and **RA** is the matrix of the transformed explanatory variables. It can be seen that **RA** also has full column rank provided that **R** is nonsingular. The solution is
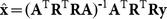
(16)


Obviously, we can see that 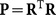. The natural question then is how to find a transformation matrix that yields the most efficient estimator among all linear unbiased estimators. Generally speaking, one should choose **R** as a non-stochastic and non-singular matrix like 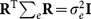. It should be note that 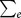 is symmetric and positive definite so that it can be orthogonally diagonalized as 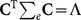, where **C** is the matrix of eigenvectors corresponding to the matrix of eigenvalues 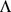. For 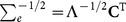, we have 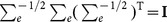. This result suggests that the transformation matrix **R** should be proportional to 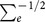. Given this choice of **R**, we have 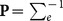.

The matrix **Q** can be estimated by using the inverse covariance matrix of **x** [30], [45]. Q is introduced to generate a Mahalanbios distance based regularization term. The main difference between ridge regression and the proposed method is that ridge regression uses Euclidean distance to constrain the representation coefficients and the proposed method applies Mahalanbios distance to constrain them. It's believed that Mahalanbios distance might provide a better regularization term than Euclidean distance since there exists correlations between representation coefficients. Ideally, we should maximize the correlation of representation coefficients of the homo-class samples and minimize the correlation of representation coefficients of the hetero-class samples simultaneously in the training process. However, it is difficult to model this because we have different numbers of representation coefficients corresponding to homo-class and hetero-class samples. A feasible way is to eliminate the correlations of all representation coefficients. This leads to more significant effects on the representation coefficients of hetero-class samples than on those of homo-class samples, since the representation coefficients of hetero-class samples are much more than those of the homo-class samples in multi-class classification problems.

Based on the above analysis, we give the details of estimating **P** and **Q** as follows.

Let 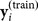 be the *i*-th sample of the training set. 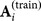 is the matrix formed by the *K* nearest neighbors of 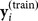 from the training set. We set **P**_0_ **= I** and **Q**_0_ **= I**. The coding coefficient vector of 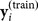 onto 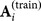 is: 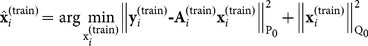
(17)


Let 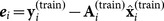 and 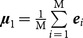. Then **P** can be estimated by 
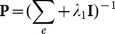
(18) where 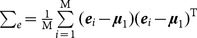. 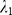 is the regular parameter. Note that 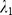 **I** is introduced to avoid the singularity of the covariance matrix.

Let 
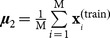
. Then, **Q** can be estimated by 
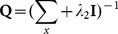
(19)where 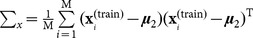. 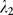 is the regular parameter and 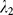 **I** is also used to avoid the singularity of the covariance matrix.

In the testing stage, for a given test sample **y**, we find its *K* nearest neighbors from the training set to form the matrix **A**. Then, we calculate the representation coefficients vector 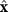 using Eq. (14). We can reconstruct the test sample **y** as 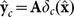 by employing the representation coefficients associated with *c*-th class. The corresponding reconstruction error of *c*-th class is defined: 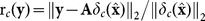
(20)


The decision rule is: if 
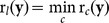
, **y** is assigned to Class *l*.

B-GRR makes full use of the prior information of the training set. It works well when the testing samples share the same probability distribution with the training samples. The algorithm of B-GRR for classification is summarized in Algorithm 1.

#### Algorithm 1

B-GRR for Classification

**Input**: Dictionary **A**, test sample **y**. Initial values **P**_0_ and **Q**_0_

1. Normalize the columns of **A** to have unit L_2_-norm.
2. The prior information matrices **P** and **Q** are learned from training set by using Eq. (18) and Eq. (19).
3. The test sample **y** is coded on its *K* nearest neighbors **A** via Eq. (12).
4. Compute the residuals of each class.

**Output**: **y** is assigned to the class which yields the minimum residuals.

### B. Robust GRR

In image classification problems, illumination, expression or pose changes may cause significant differences between test samples and training samples. Therefore, it is necessary to introduce the test sample specific information to alleviate the effect caused by the differences between test samples and training samples. This specific information is to give a weight to each feature (or image pixel) of the sample, which can be learned online via the iteratively reweighted algorithm.

Based on this idea, we present a robust general regression and representation model (R-GRR) for classification. Compared with B-GRR, R-GRR not only includes the prior information **P** and **Q**, but also contains the specific information (weight matrix) **W**. The model is given below: 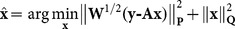
(21)


If **P**, **Q** and **W** are known, the above model can be solved explicitly using the formula: 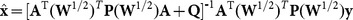
(22)


Since **P** and **Q** can be learned offline using the same method as in Basic GRR, the remaining problem is to learn the specific information **W** online. Specifically, given a test sample **y**, we firstly compute the representation residuals ***e*** of **y** so as to initialize the weight. The residual ***e*** is initialized as ***e*** = **y**-**y** *_ini_*, and **y** *_ini_* is the initial estimation of the true images from the observe samples. In this study, we simply set **y** *_ini_* as the mean image of all samples in the coding dictionary since we don't know which class the test image **y** belongs to. With the initialized **y** *_ini_*, our method can estimate the weight matrix **W** iteratively. **W** actually is a diagonal matrix, **W** *_k_*_,*k*_ (i.e. 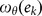) is the weight assigned to the *k*-th pixel of test image. The weight function [16] is:
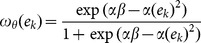
(23)where 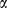 and 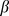 are positive scalars.

In addition, Eq. (22) is the explicit solution of Eq. (21). The process is terminated when the difference of the weights between adjacent iterations satisfies the following condition:
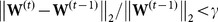
(24)


The R-GRR algorithm for classification is summarized in Algorithm 2.

#### Algorithm 2

R-GRR for Classification

**Input**: Dictionary **A**, test sample **y**. Initial values **P**_0_, **Q**_0_ and **y** *_ini_*.

1. Normalize the columns of **A** to have unit L_2_-norm, test sample y with L_2_-norm and y*^t^* initialized as y*_ini_*.
2. The prior information matrices **P** and **Q** are learned from the training set by using the generalized Tikhonov regularization and KNN.
3. The test sample **y** is coded on its *K* nearest neighbors **A**.
  - Compute residual 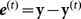

  - Estimate weights 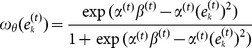

  - Code 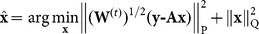

  - Compute the reconstructed test sample 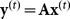, and let t  =  t + 1
  - Go back to step a) until the maximal number of iterations is reached, or convergence is met as shown in Eq. (24)
4. Compute the residuals of each class.

**Output**: **y** is assigned to the class which yields the minimum residuals.

### C. Robust GRR for Occlusion Cases

In real-world image recognition tasks, occlusion is one of the most challenge problems. To overcome this problem, we combine advantages of the prior information **Q** and the specific information **W** to enhance the classification performance. As we know, **P** reflects the correlations between representation residuals. If there are great differences between the test sample and the training samples, the resulting reconstruction error does not follow the original distribution. In this case, we cannot employ the matrix **P** to eliminate the correlations between representation residuals of test images. So, the matrix **P** is removed from the model when there is occlusion, real-disguise or noises in test image. In contrast, the matrix Q won't be affected whether the test image has occlusion or not since it is mainly used as a regularization term. Therefore, we keep matrix **Q** in the model, which can have positive effect on the performance. Then Eq. (19) can be reformulated as: 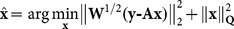
(25)


The solution of this model is: 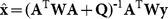
(26)


## Further Analysis of GRR

**Figure 3:**
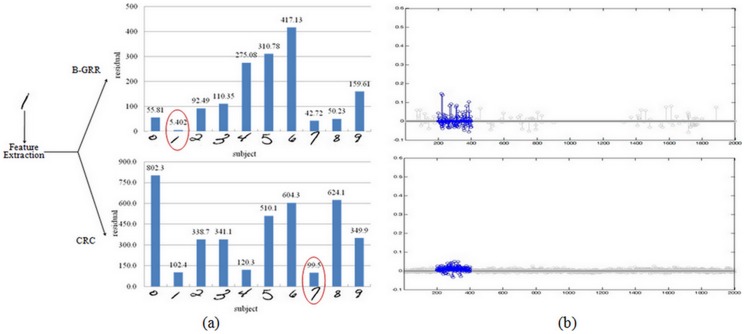
The example shows the importance of P and Q for classification. (a) The classification result is right when our model B-GRR using the prior information **P** and **Q**. However, we obtain the wrong result using CRC. (b) The corresponding representation coefficients of B-GRR and CRC.

In this section, we will further analyze the role of **P** and **Q** in GRR. **P** is a symmetric matrix which is learned from the training set and can be decomposed into 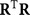, where **R** is a non-singular transformed matrix and is used to eliminate the correlations between representation residuals. The matrix **Q** in the regularization term is also learned from the training set. The proposed model uses Mahalanbios distance instead of Euclidean distance to constrain the representation coefficient. It's believed that Mahalanbios distance can provide a better regularization than Euclidean distance since there exists correlations between representation coefficients. Fig. 3(a) gives an example to show the role of **P** and **Q**. In this example, we represent the test sample “1” from the CENPARMI database and illustrate the reconstruction residual of each class. Based on the minimal class residual criterion, we know that B-GRR, using the prior information contained in **P** and **Q**, achieves the right result, while CRC fails without using this information.

We then compare the obtained representation coefficients of CRC and B-GRR. Fig. 3 (b) shows the representation coefficients of CRC and B-GRR for the same test image as shown in Fig. 3 (a). The representation coefficients of the homo-class samples are highlighted in blue. CRC provides a very dense representation, while B-GRR gives a sparser representation due to the KNN based dictionary selection. In comparison with CRC, the representation coefficients of B-GRR seem to be more congregated on the homo-class samples.

**Figure 4:**
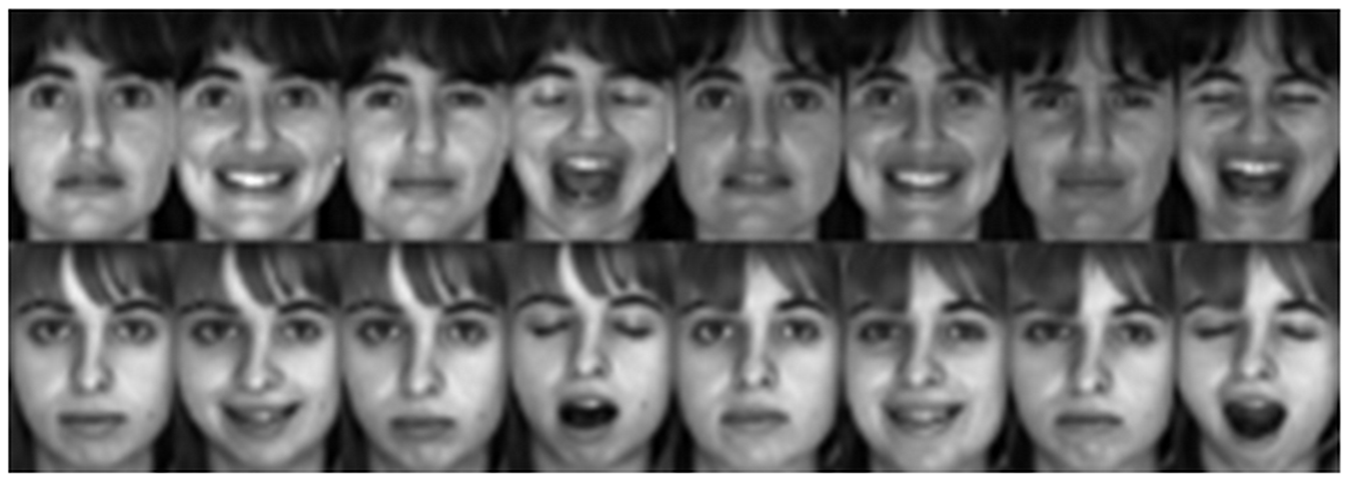
Two classes of samples from the AR database. (a) Recovered clean image and occluded part via six methods for the image with sunglasses. For R-GRR, RRC_L2 and RSC, we also give the corresponding weight maps. (b) Recovered clean image and occluded part via six methods for the image with scarf. For R-GRR, RRC_L2 and RSC, we also give the corresponding weight maps.

**Figure 5:**
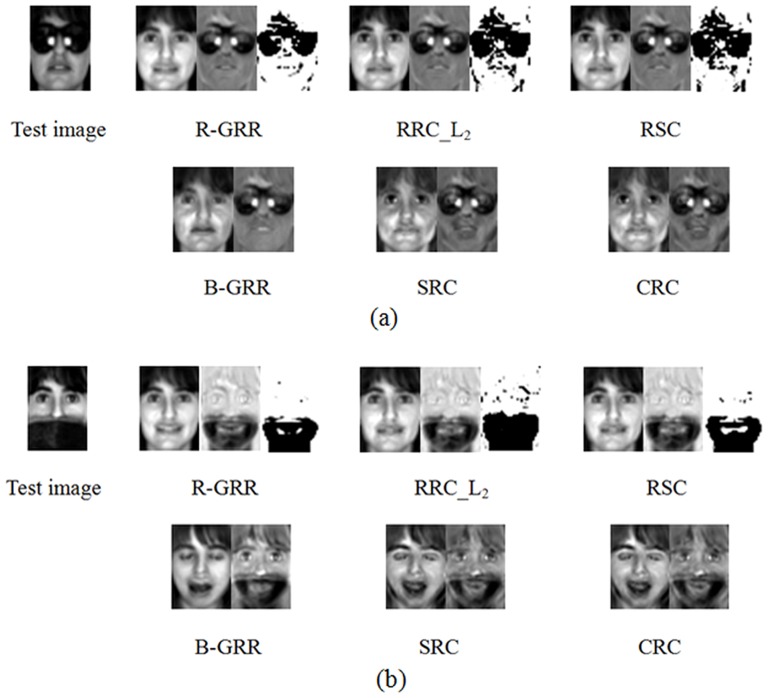
Examples for handing occlusion images.

We also give an example to compare our methods with some state-of-the-art methods on handing occlusions. In the example, two classes of face images from the AR database, as shown in Fig. 4, are used for training. We test two cases of real-world disguise images: the images with sunglasses and the images with scarves. In Fig. 5 (a) and Fig. 5 (b), the left column contains the disguise images. In our test, we use R-GRR, RRC_L_2_, RSC, B-GRR, SRC and CRC to deal with occlusion. For each occluded image, the reconstructed images (recovered clean image) and the residual images (recovered occlusion) are shown in Fig. 5. From Fig. 5, we can see that R-GRR achieves comparable result with RRC_L_2_, RSC and significantly outperforms other methods. However, R-GRR is slightly better than RRC_L_2_ and RSC from the viewpoint of weight maps.

## Experiments

In this section, we perform experiments on four benchmark databases and compare the proposed model GRR with state-of-the-art models. Note that here in SRC and RSC, the matlab function “l1-ls” [25] is used to calculate the sparse representation coefficient. In the following experiments, the parameter 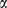 is 8 and 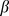 is 0.8 for image classification. 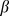 is set to 0.5 when dealing with occlusion cases [16].

### A. Handwritten Numeral Recognition

#### CENPARMI Database

**Figure 6:**
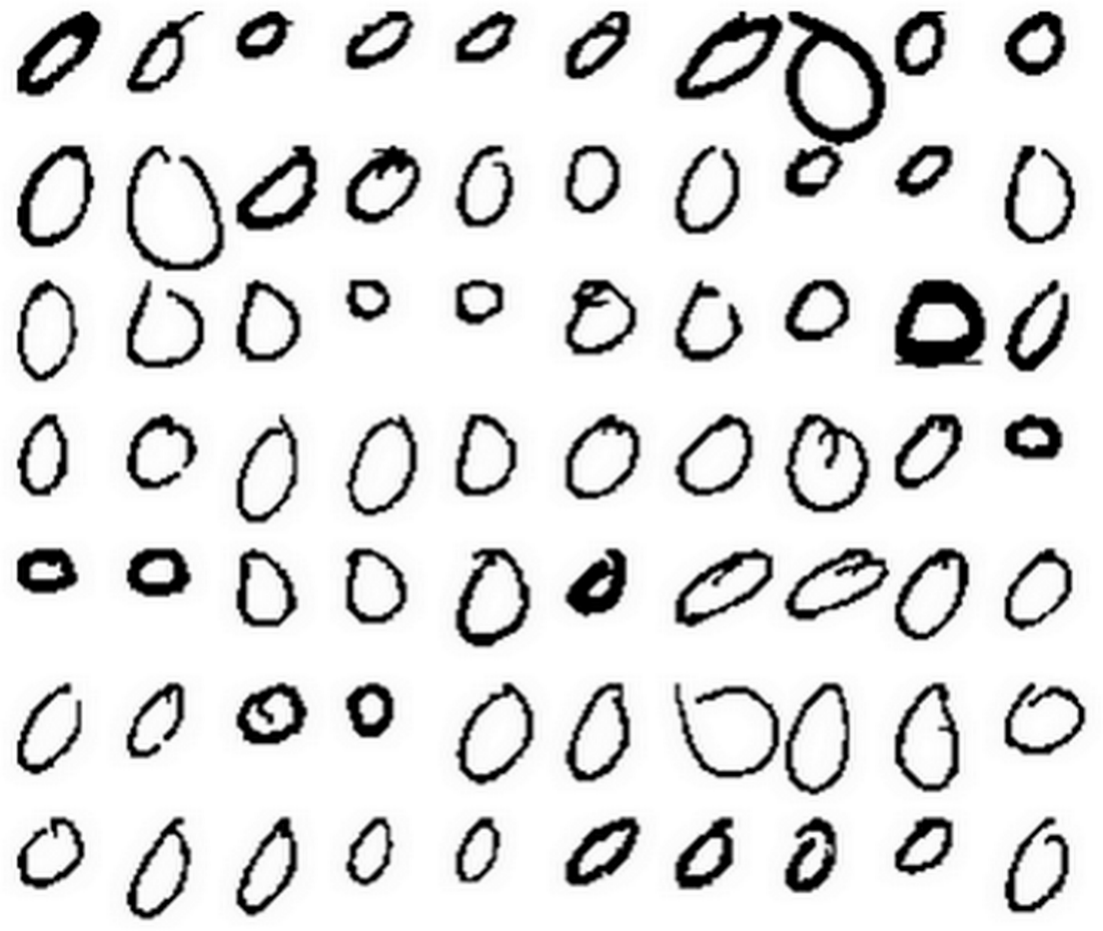
Some samples in CENPARMI database.

The experiment was done on Concordia University CENPARMI handwritten numeral database. The database contains 6000 samples of 10 numeral classes (each class has 600 samples). Some samples of “0” from the CENPARMI database are shown in Fig. 6.

**Figure 7:**
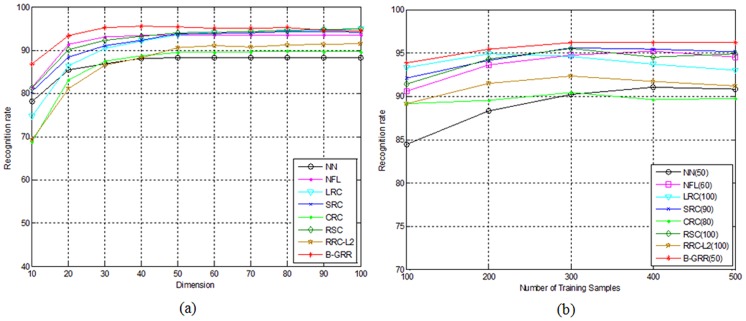
(a) The recognition rate of each method for handwritten numeral recognition on the CENPARMI database versus the variation of dimensions; (b) The recognition rate of each method corresponds to the number of classs training samples that varies from 100 to 500 with an inerval 100. The number in brakets of each method means the number of low dimentional trasforamation features.

In the first experiment, we choose the first 200 samples of each class for training, the remaining 400 samples for testing. Thus, the total number of training samples is 2000 while the total number of testing samples is 4000. PCA is used to transform the original 121-dimensional Legendre moment features [28] into D-dimensional features, where D varies from 10 to 100 with an interval 10. Based on the PCA-transformed features, NN, NFL, LRC, SRC, CRC, RSC, RRC_L_2_ and B-GRR are employed for classification. The parameter K is set to 200. The recognition results of each method corresponding to the variation of dimensions is shown in Fig. 7 (a). From Fig. 7 (a), we can see that B-GRR achieves the best results among all competing methods. The maximal recognition rate of B-GRR is 95.4%, compared to 88.3% for NN, 93.6% for NFL, 94.9% for LRC, 89.7% for CRC, 94.3% for SRC, 89.7% for CRC, 95.0% for RSC and 91.5 for RRC_L_2_. LRC gives the comparable results since there are enough training samples in each class.

In the second experiment, we let the number of training samples per class vary from 100 to 500 with an interval of 100, and the rest samples for testing. Then, PCA is used to transform the original Legendre moment features into low-dimensional features. We select the optimal dimension of each method based on the above experiments as shown in Fig. 7 (b). The recognition rates of each method corresponding to the variation of training samples is shown in Fig. 7 (b). From Fig. 7 (b), we can see that B-GRR still gives better results than other competing methods.

#### NUST603 Database

**Figure 8:**
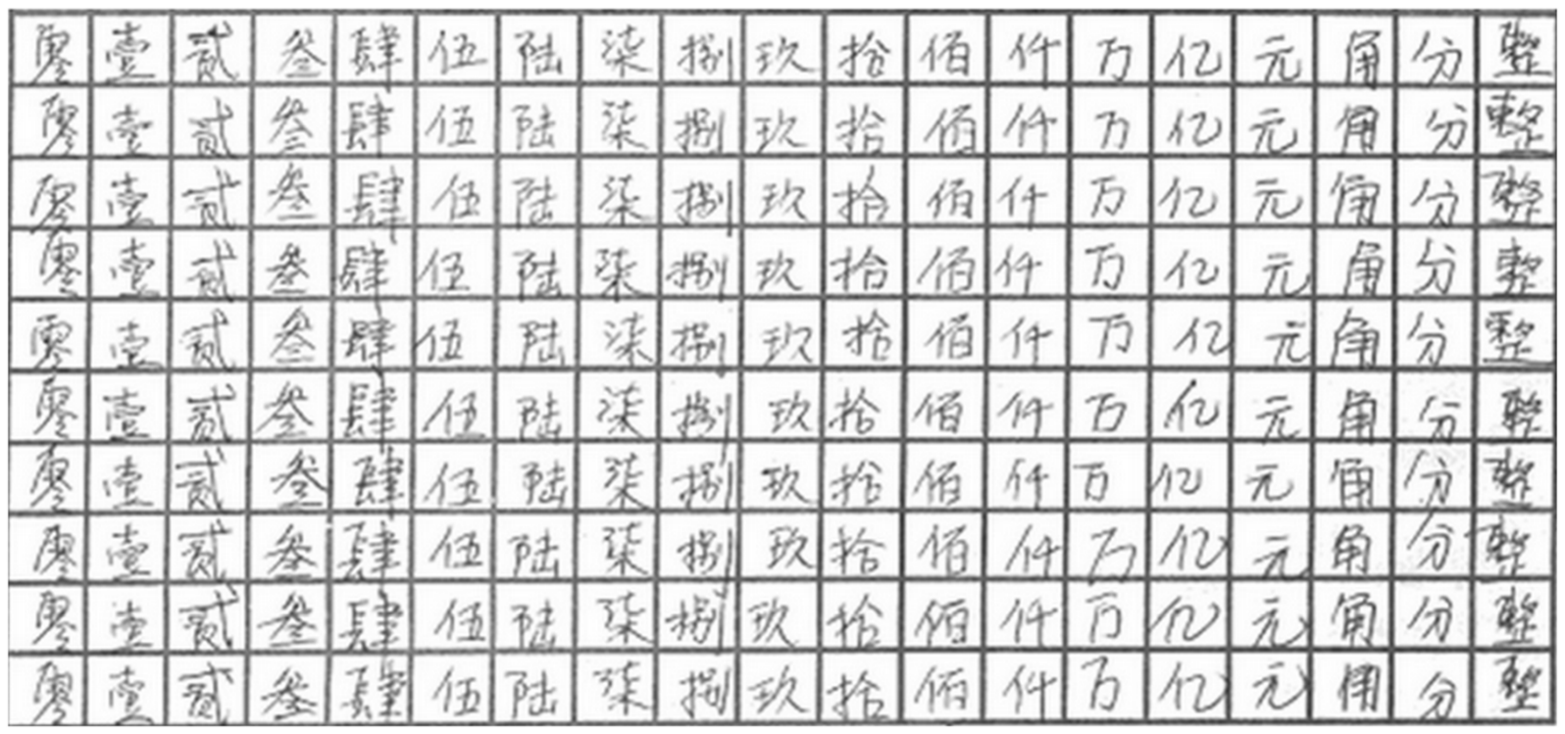
Some samples in NUST603HW database.

The experiment was performed on the NUST603 handwritten Chinese character database which was built in Nanjing University of Science and Technology. The database contains 19 groups of Chinese characters that are collected from bank checks, each group with 400 samples. Some images from the NUST603HW database are shown in Fig. 8.

**Figure 9:**
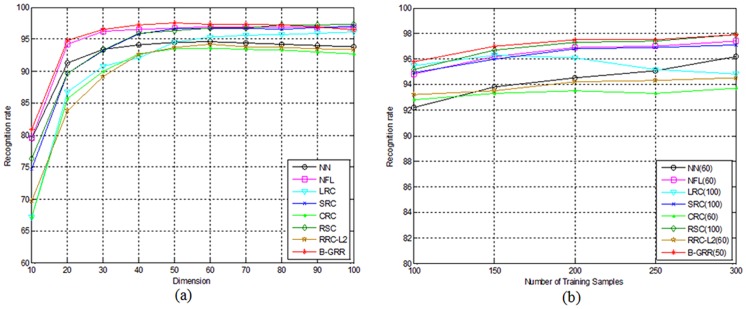
(a) The recognition rate of each method for handwritten numeral recognition on the NUST603 database versus the variation of dimensions; (b) The recognition rate of each method corresponds to the number of classs training samples that varies from 100 to 300 with an inerval 50. The number in brakets of each method means the number of low dimentional trasforamation features.

In this experiment, the first 200 samples of each class are used for training, and the remaining samples for testing. Similar to the experimental methodology adopted in the last experiment. PCA is used to transform the original 128-dimensional peripheral feature [29] into D-dimensional features. We thus let D varies from 10 to 100 with interval 10. The parameter K is set to 300. Then NN, NFL, LRC, SRC, CRC, RSC, RRC_L_2_ and B-GRR are employed for classification. The performances of each method versus the variation of dimensions are shown in Fig. 9 (a). Additionally, we also let the number of training samples per class vary from 100 to 300 with an interval of 50, and the remaining samples for testing. PCA is then used to transform the original feature into low-dimensional features. We select the optimal dimension of each method based on the above experiments as shown in Fig. 9 (b). The recognition rates of each method are illustrated in Fig. 9 (b). The results in Fig. 9 are basically consistent with those in Fig. 7. B-GRR still achieves the better performance than other methods, irrespective of the variation of dimensions or training sample sizes.

### B. Face Recognition without Occlusion

We evaluate the performance of R-GRR on the AR and the Extended Yale B database with illumination and expression changes but without occlusion. In these experiments, PCA is first used to reduce the dimensionality of face image.

#### AR Database

The AR face database [26] contains over 4000 color face images of 126 persons, including frontal views of faces with different facial expression, lighting conditions and occlusions. The pictures of 120 individuals were taken in two sessions (separated by two weeks) and each session contains 13 color images. Fourteen face images (each session contains 7) of 100 individuals are selected and used in our experiment. The face portion of each image is manually cropped and then normalized to 60_×_43 pixels.

In this experiment, images from the first session are used for training, and images from the second session are used for testing. Then NFL, LRC, SRC, CRC, B-GRR, RSC, RRC_L_2_ and the proposed R-GRR are employed for classification. The NN classifier is also used to provide a baseline. The parameter K of R-GRR means we choose the K nearest neighbors of the test image from training set to form the coding dictionary. K is set to 650 here. The recognition rates of each classifier versus the variation of dimensions are listed in Table 1. From Table 1, we can see that our model R-GRR outperforms state-of-the-art methods in all dimensions except that R-GRR is slightly worse than RSC when dimension is 54. However, it's difficult to achieve better performance when dimension is low for all the methods. The maximal recognition rates of NN, NFL, LRC, SRC, CRC, B-GRR, RSC, RRC_L_2_ and R-GRR are achieved when the dimension is 300.

**Table 1:** The recognition rate of each classifier for face recognition on the AR database.

Dim	54	120	300
NN	68.0	70.1	71.3
NFL	69.2	72.7	73.4
LRC	71.0	75.4	76.0
SRC	83.3	89.5	93.3
CRC[23]	80.5	90.0	93.7
B-GRR	81.3	90.4	93.6
RSC[16]	***86.8***	94.0	96.0
RRC_L_2_ [31]	84.3	94.3	95.3
R-GRR	85.6	***95.3***	***97.3***

#### Extended Yale B Database

The extended Yale B face image database [27] contains 38 human subjects under 9 poses and 64 illumination conditions. The 64 images of a subject in a particular pose are acquired at camera frame rate of 30 frames/second, so there are only small changes in head pose and facial expression for those 64 images. All frontal-face images marked with P00 are used in our experiment, and each is resized to 48×42 pixels.

In our experiment, we use the first 32 images of each individual for training and the remaining images are used for testing. Based on the PCA-transformed features, NN, NFL, LRC, SRC, CRC, B-GRR, RSC, RRC_L2 and R-GRR are employed for classification. The parameter K is 800. The recognition rates of each classifier corresponding to the variation of feature dimensions are listed in Table 2. Table 2 shows that the proposed model R-GRR achieves the best recognition results in all dimensions for face recognition. When the feature dimension is 100, R-GRR gives about 3% improvement of recognition rate over LRC, SRC and CRC, respectively.

**Table 2:** The recognition rate of each classifier for face recognition on the Extended Yale B database.

Dim	50	100	200
NN	78.5	85.8	90.2
NFL	88.7	90.5	91.0
LRC	93.3	94.8	95.2
SRC	93.7	94.7	95.6
CRC	91.9	94.7	96.5
B-GRR	92.1	94.9	97.1
RSC	94.2	97.0	98.2
RRC_L_2_	93.4	97.0	98.3
R-GRR	***94.3***	***97.6***	***98.4***

### C. Face Recognition with Occlusion

In this section, we examine the robustness of R-GRR when face images suffer different occlusions, such as real disguise, block occlusion or pixel corruption. In the following experiments, we mainly compare our method with CRC, SRC, RSC, RRC_L_2_, correntropy-based sparse representation (CESR) [17] and Gabor-SRC [13].

#### Face Recognition with Real Disguise

A subset of the AR face image database is used in our experiment. The subset includes 100 individuals, 50 males and 50 females. All the individuals have two session images and each session contains 13 images. The face portion of each image is manually cropped and then normalized to 42×30 pixels.

In the first experiment, we choose the first four images (with various facial expressions) from the session 1 and session 2 of each individual to form the training set. The total training images is 800. There are two image sets (with sunglasses and scarves) for testing. Each set contains 200 images (one image per session of each individual with neutral expression). The parameter K is 300 for the test set with sunglasses and 760 for the test set with scarves. The face recognition results of each method on the two testing set are listed in Table 3. From Table 3, we can see that R-GRR achieves the best recognition results among all the methods when the images with scarves and gives comparable result with the excellent method when the images with sunglasses. Additionally, the performances of RRC_L_2_, RSC and CESR are higher when facial image with sunglasses. However, CESR only achieves 42% when facial images with scarves.

**Table 3:** The recognition rate of each classifier for face recognition on AR database with disguise occlusion.

Methods	Sunglasses	Scarves
CRC	65.5	88.5
SRC	87.0	59.5
GSRC[13]	93.0	79.0
CESR[17]	99.0	42.0
RSC[16]	99.0	97.0
RRC_L_2_ [31]	***99.5***	96.5
R-GRR	***99.5***	***99.0***

**Figure 10:**
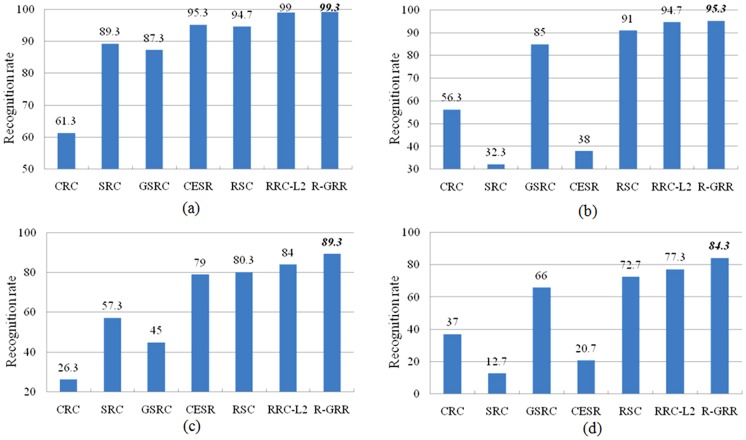
The recognition rates of each classifier for face recognition on AR database with disguise occlusion. (a) The testing images with sunglasses from session 1; (b) The testing images with scarves from session 1; (c) The testing images with sunglasses from session 2; (d) The testing images with scarves from session 2.

In the second experiment, four neutral images with different illumination from the first session of each individual are used for training. The disguise images with various illumination and glasses or scarves per individual in session 1 and session 2 for testing. We set the parameter *K* as 220, 300, 240 and 320 for the four different test sets, respectively. The recognition rates of each method are shown in Fig. 10. From Fig. 10, we can see clearly that R-GRR gives better performance than CRC, SRC, GSRC, CESR, RSC and RRC_L_2_ on different testing subsets. Both SRC and CESR do well on the subsets with sunglasses but poor in the cases with scarves. However, GSRC achieves better result on the subsets with scarves and worse result on the subsets with sunglasses. Compared to RSC, at least 4.3% improvement is achieved by R-GRR for different testing set. Meanwhile, it is worth noticing that the recognition rate of R-GRR is 67.6%, 59.6% higher than SRC and CESR on the testing images with scarves from session 2, and 43.7% higher than GSRC on the testing images with sunglasses from session 2. In the first two subsets from session 1, the performances of R-GRR and RRC_L_2_ are similar. However, R-GRR significantly outperforms RRC_L_2_ in the last two subsets (more challenge tasks) from session 2. Compared with RRC_L_2_, R-GRR uses 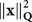 instead of 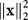 to refine the regularization term can further improve the classification performance.

#### Face Recognition with Block Occlusion

**Figure 11:**
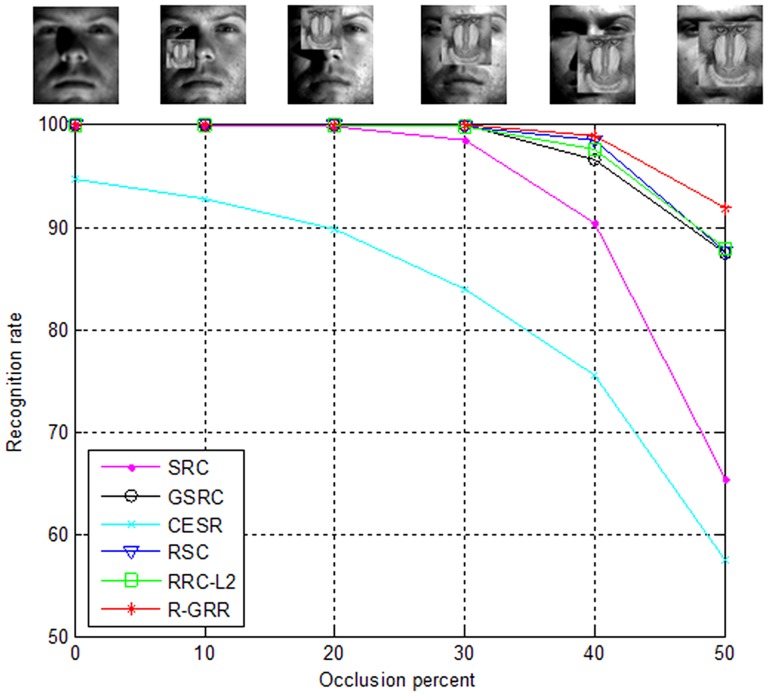
The recognition rates of SRC, GSRC, CESR, RSC, RRC_L_2_ and R-GRR under the occlusion percentage from 0 to 50.

In this experiment, we use the same experiment setting as in [12], [16] to test the robustness of R-GRR. Subsets 1 and 2 of the Extended Yale B database are used for training and Subset 3 is used for testing. The face images are resized to 96×84. The parameter K is 500. Fig. 11 shows recognition rates curve of SRC, GSRC, CESR, RSC, RRC_L_2_ and R-GRR versus the various levels of occlusion (from 0 percent to 50 percent). From Fig. 11, we can see that the proposed R-GRR overall outperforms SRC, GSRC, CESR, RSC and RRC_L_2_. When the occlusion percentage is 50%, R-GRR achieves the best recognition rate 91.9, compared to 65.3 for SRC, 87.4 for GSRC, 57.4 for CESR, 87.6 for RSC, and 87.8 for RRC_L_2_. It's surprising that the performance of CESR is very poor. Probably, it is not suitable for dealing with this block occlusion case.

#### Face Recognition with Pixel Corruption

**Figure 12:**
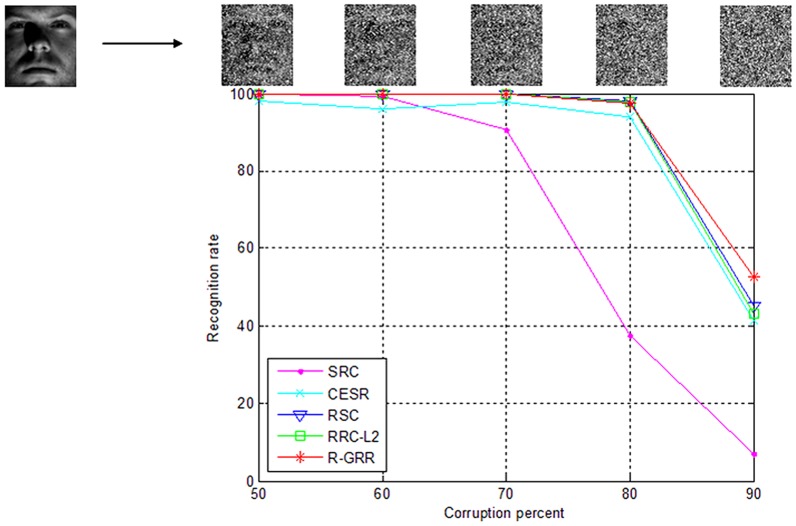
The recognition rates of SRC, GSRC, CESR, RSC, RRC_L_2_ and R-GRR under the pixel corruption percentage from 50% to 90%.

In this experiment, we chose the images from the Subsets 1 and 2 of the Extended Yale B database for training, and images from the Subset 3 with random pixel corruption (the image is corrupted by using uniformly distributed random values within [0, 255]) for testing. The face images were resized to 96×84 pixels. The corrupted pixels are randomly chosen for each test image and the locations are unknown to the algorithm. We vary the percentage of corrupted pixels from 0% to 90%. Since the most competing methods can achieve better performance from 0% to 40%. We only report the recognition rates for 50%**–**90% corruption. Fig. 12 plots the recognition rates of five methods under different levels of corruptions. From Fig. 12, we can see that R-GRR, RRC-L_2_, and RSC give the similar results in 80%, 70%, 60% and 50% corruption. R-GRR achieves the best recognition rate when the percentage of corrupted pixels is 90%. However, the performance of SRC is poor when the percentage of corrupted pixels is more than 70%.

### D. Discussion

In this section, we first discuss the influences of the parameters K, 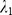 and 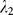 in our experiments. We then compare the running time of the proposed R-GRR with state-of-the-art methods.

**Figure 13:**
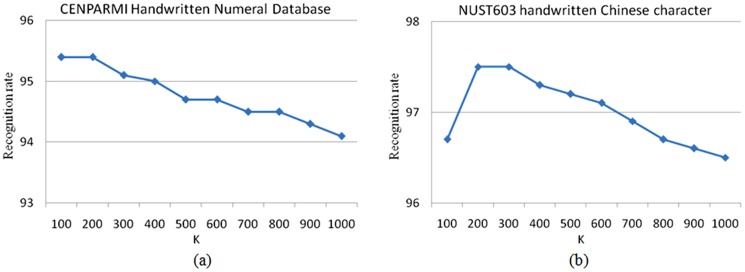
The recognition rate curves of B-GRR versus the variation of parameter K in handwritten numeral recognition tasks. (a) Experiment on the CENPARMI database; (b) Experiment on the NUST603 database.

**Figure 14:**
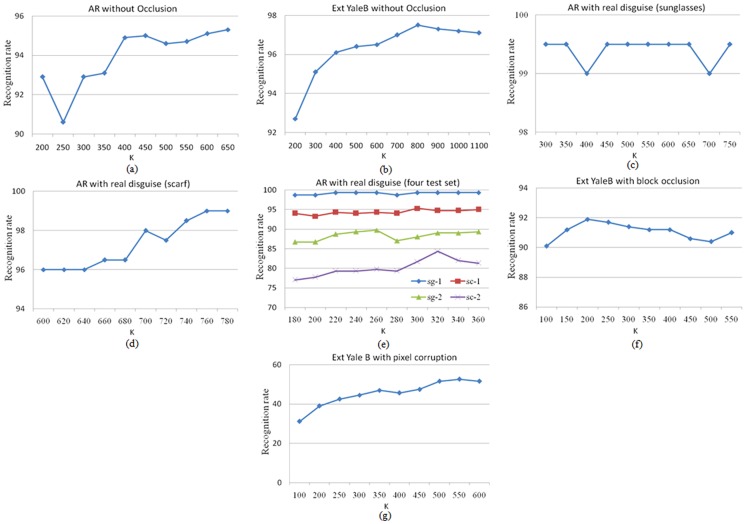
The recognition rate curves of R-GRR versus the variation of parameter K on the different experiments. (a) the images without occlusion for test; (b) the images without occlusion for test; (c) the images with sunglasses for test; (d) the images with scarf for test; (e) the images with sunglasses (sg-X) or scarf (sc-X) in session X for test; (f) the images with block occlusion (50%) for test; (g) the images with pixel corruption (90%) for test.

The performances of the proposed method R-GRR (or B-GRR) with different parameters are evaluated on different recognition scenarios. The experiments setting are same with the above mentioned experiments in section 5.2 and 5.3. In our experiments, we just change one parameter when fixing the other ones. Fig. 13 plots the recognition rates versus the variation of the parameter K on the CENPARMI database and NUST603 database. From Fig. 13, we can see that B-GRR can achieve the better recognition rates in conjunction with a smaller K. Fig. 14 plots the recognition rates versus the variation of the parameter K in different face recognition experiments. From Fig. 14 (a) and (b), we can see that the parameter K is relatively larger and smaller than total number of training samples will lead to higher performance when face images without occlusion. Fig. 14 (c) and (e) show that the recognition rates are not sensitive to the variations of the parameter K. In Fig. 14 (f), the proposed method achieves best results when the K is 200 for the test images with block occlusion. However, R-GRR gives the best performance when the K is set to 550 for the test images with pixel corruption as shown in Fig. 14 (g). Generally speaking, the parameter K is relatively smaller in the case that the feature dimension is much lower than the number of training samples, while the parameter K is relatively larger in the case that the feature dimension is much higher than the number of training samples. In this paper, we employ the cross-validation strategy to determine the parameter K in the training stage. Specifically, we select one training sample as query sample and the rest training samples as gallery set. Thus, the recognition rate of all training samples can be achieved. We choose the best parameter K which achieves the best recognition rate.

**Figure 15:**
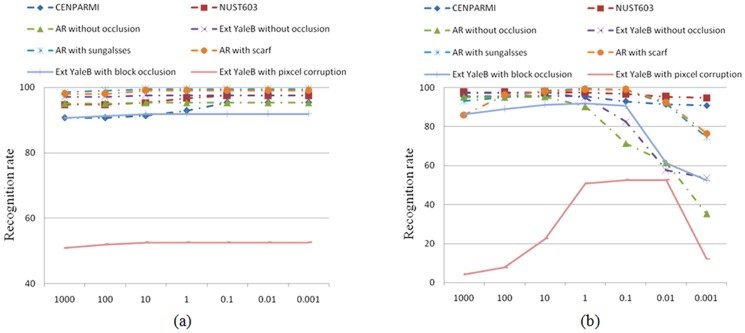
The recognition rate curves of the proposed model versus the variation of regularization parameters on the different experiments. (a) The influence of 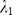. (b) The influence of 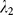.

Fig. 15 plots the recognition rates versus the variation of the regularization parameters 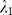 and 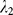, respectively. From Fig. 15, we can see that the proposed model always achieves it optimal or nearly optimal performance when 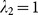 under different face recognition scenarios. However, the performance of the proposed model is non-sensitive to the variation of 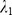. Thus, it's easy to set the regularization parameters of the proposed methods in real-world applications.

The running time of the competing methods, including SRC, GSRC, CESR, RSC, RRC_L_2_ and R-GRR, are evaluated on the AR database (with sunglasses). The programming environment is Matlab version 11b. The desktop used is of 2.93 GHz CPU and with 4G RAM. Table 4 lists the computation time for one recognition operation of various methods with the same experiment setting in Section 5.3.1. Note that we only compute the online running time for R-GRR. From Table 4, we can see that R-GRR is superior to SRC, GSRC and RSC due to less computation cost and better performance. RRC_L_2_ achieves the least computation time. SRC has rather high computation burden. In addition, RSC is very time-consuming since it must solve L_1_ optimization problem in each iteration process. Although CESR is also fast, its performance is not stable. R-GRR gives comparable computation time with RRC_L_2_ and achieves better performance than RRC_L_2_ in most cases.

**Table 4:** Computation time of R-GRR and state-of-the-art methods.

Methods	Time (s)
SRC	96.5
GSRC	12.3
CESR	0.36
RSC	142.9
RRC_L_2_	0.21
R-GRR	0.24

## Conclusions and Future Works

In this section, we first conclude the paper and then give more discussions on potential future work.

This paper presents a general regression and representation (GRR) model for pattern classification. In GRR, we learn the prior information from the training set by using the generalized Tikhonov regularization and KNN, and obtain the specific information from the test sample by using the iteratively reweighted algorithm. Actually, we provide two classifiers: B-GRR and R-GRR, which combine the prior information and the specific information with different strategies. Experiments on character datasets and face datasets demonstrate that the validity of our model and its performance advantages over state-of-the-art classification methods. Particularly, R-GRR achieves encouraging recognition rates under different cases but with lower computational cost.

Although our model has demonstrated promising performance, there are still many issues requiring in-depth investigation in the future. Here, two improvements can be made for GRR. (1) Most classification methods perform well on the condition that they assume the training and testing data are drawn from the same feature space and the same distribution. However, it's difficult to hold this assumption in real-world applications. To address this problem, transfer learning is proposed and aims to help improve the target predictive function using the knowledge in source domain [50]. Deng et al. presented the generalized hidden-mapping ridge regression method for various types of classical intelligent methods [51]. We can borrow the idea of transfer learning to improve the robustness of our model. (2) With the ever increasing size of training data sets, a challenge in our model is how to design an efficient learning algorithm. Actually, there are many literatures have been reported to overcome the similar problem. IvorW. Tsang et al. presented a core vector machine (CVM) to handle larger datasets. Furthermore, CVM not only preserves the performance of SVM but also performs much faster than existing scale-up methods [52]. Deng et al also developed effective learning algorithms for fussy models when facing with large datasets [53], [54].

### Ethics Statement

Some face image datasets were used in this paper to verify the performance of our methods. These face image datasets are publicly available for face recognition research, and the consent was not needed. The face images and the experimental results are reported in this paper without any commercial purpose.
